# Supplementary material for: Trisomies Reorganize Human 3D Genome
Source: Int J Mol Sci. 2023 Nov 7;24(22):16044. doi: 10.3390/ijms242216044 (PMC10671006; doi:10.3390/ijms242216044)
Supplement: Supplementary file 1 [file ijms-24-16044-s001.zip › Figure S1.pdf]

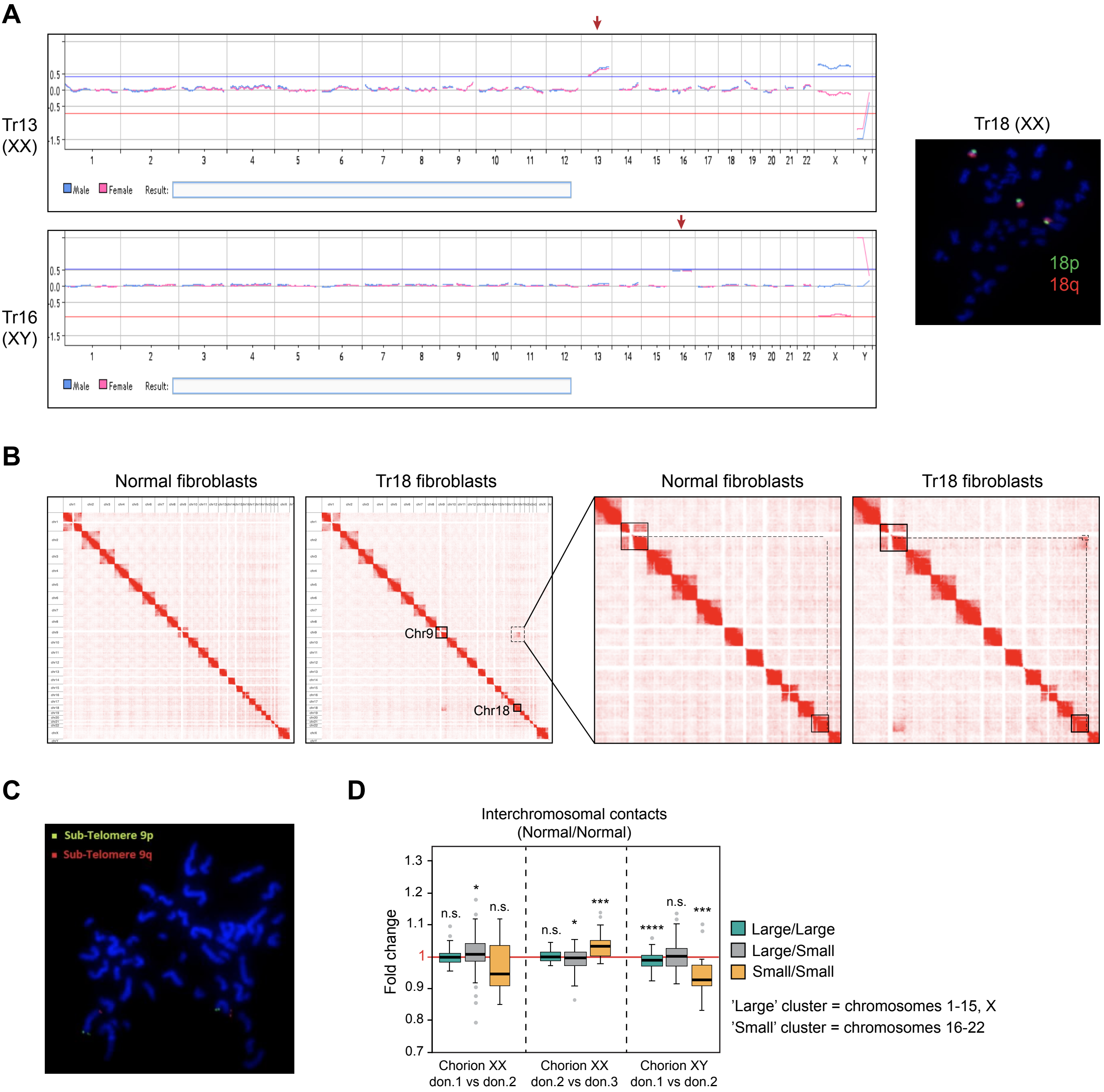

Supplementary Figure S1. Karyotype analysis and interchromosomal contacts in normal cells. (A) Karyotype analysis in Tr chorion cells (left, arrCGH) and Tr fibroblasts (right, FISH). (B) Whole-genome Hi-C maps of normal and Tr fibroblasts demonstrating the rearrangement between chr9 and chr18. (C) FISH imaging chr9 (telomeric probes) in Tr18 fibroblasts. (D) Distributions of fold change of interchromosomal number of contacts (Normal/Normal). **** - $p < 0.0001$, *** - $p < 0.001$, * - $p < 0.05$, n.s.—non-significant in a Mann–Whitney U-test
