# Supplementary material for: Trisomies Reorganize Human 3D Genome
Source: Int J Mol Sci. 2023 Nov 7;24(22):16044. doi: 10.3390/ijms242216044 (PMC10671006; doi:10.3390/ijms242216044)
Supplement: Supplementary file 1 [file ijms-24-16044-s001.zip › Figure S2.pdf]

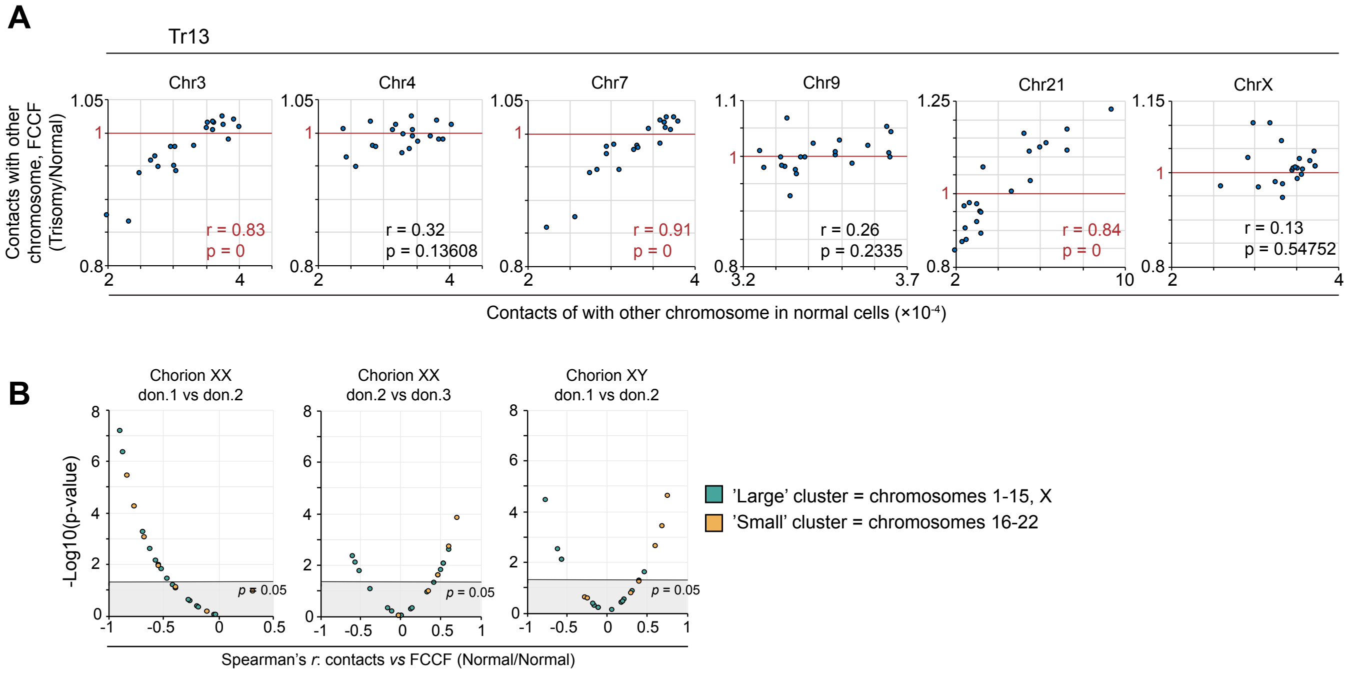

Supplementary Figure S2. Changes in contact profiles of individual chromosomes in comparisons of Tr vs. normal and normal vs. normal cells. (A) Representative examples of the dependence between contact number of a chromosome with other chromosomes in normal cells and its fold change (Tr13/normal). r—Spearman's correlation coefficient. (B) Spearman's correlation coefficient between contact number of a chromosome with other chromosomes in its fold change (Normal/Normal). Ranged by value and significance.
