# Supplementary material for: Trisomies Reorganize Human 3D Genome
Source: Int J Mol Sci. 2023 Nov 7;24(22):16044. doi: 10.3390/ijms242216044 (PMC10671006; doi:10.3390/ijms242216044)
Supplement: Supplementary file 1 [file ijms-24-16044-s001.zip › Figure S3.pdf]

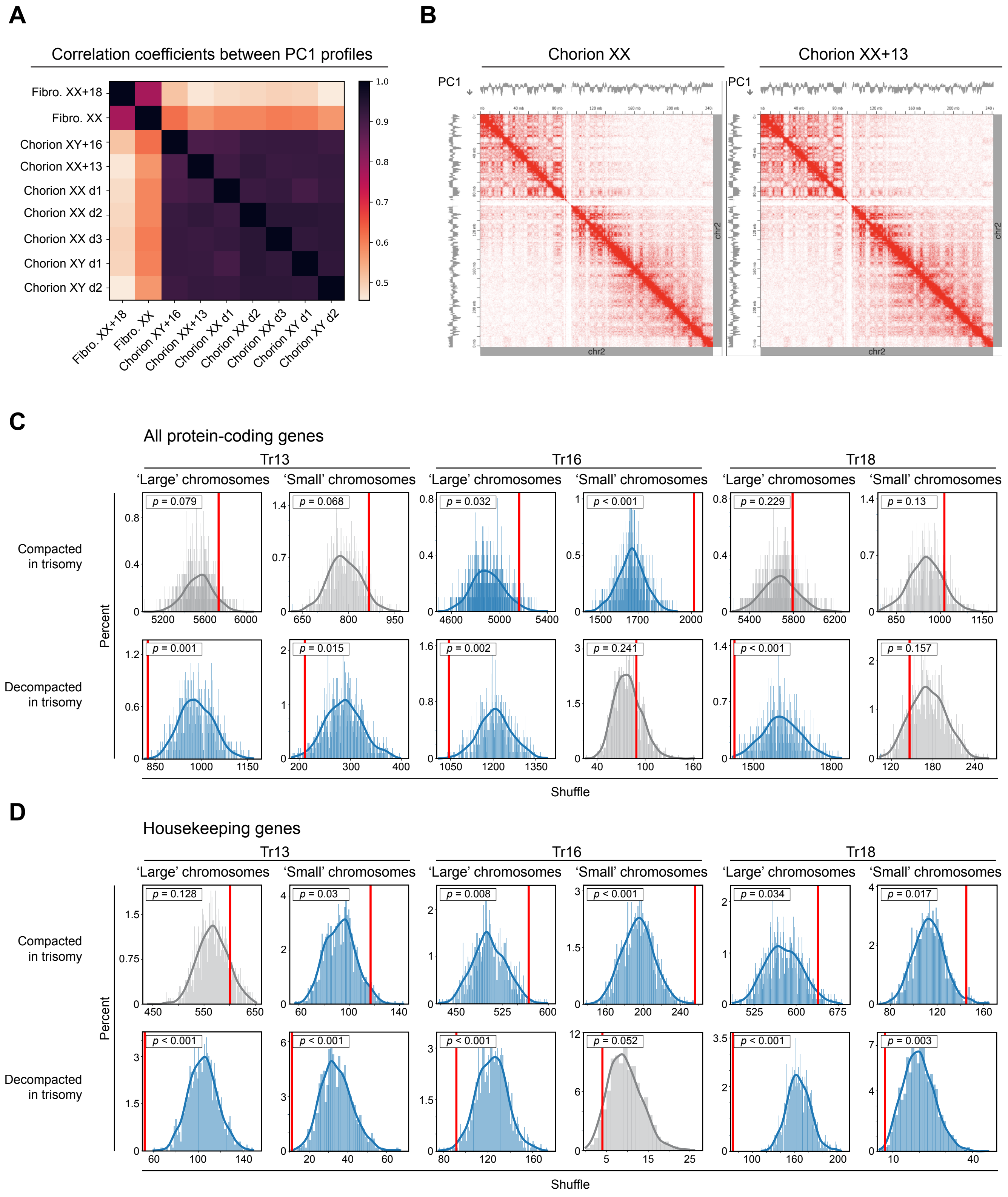

Supplementary Figure S3. Analysis of compartment profiles and gene localization. (A) Correlation between A/B compartment profiles in studied samples. Note the overall high similarity between Tr and normal karyotypes within the cell type. (B) Representative examples of the PC1 profile. (C),(D) Localization of all protein coding genes (C) and housekeeping genes (D) in loci with altered chromatin compaction.
